# The relationship between dyslipidemia and chronic liver disease, with the mediating role of depressive symptoms

**DOI:** 10.3389/fpubh.2025.1581622

**Published:** 2025-08-25

**Authors:** Wencheng Li, Youlan Zhou, Qingni Li, Deqiang Wang

**Affiliations:** Department of Infectious Diseases, Yiyang Central Hospital, Yiyang, China

**Keywords:** dyslipidemia, chronic liver disease, depressive symptoms, mediation analysis, Chinese population

## Abstract

**Background:**

Dyslipidemia and chronic liver disease (CLD) remain major global health challenges with high morbidity and mortality rates. Although extensively studied, the association between dyslipidemia and CLD remains incompletely elucidated. Depressive symptoms, an increasingly prevalent comorbidity, have been widely implicated in both conditions. This study aimed to investigate the bidirectional effects between dyslipidemia and CLD and the mediating role of depressive symptoms in their association.

**Methods:**

We recruited 6,926 participants aged ≥45 years from the China Health and Retirement Longitudinal Study (CHARLS). It used Logistic regression and mediation analysis to examine the bidirectional link between dyslipidemia and CLD, and the mediating role of depressive symptoms.

**Results:**

The median age was 58.7 years. Among participants, 222 were diagnosed with CLD and 1,883 with dyslipidemia. After adjusting for confounders, individuals with dyslipidemia exhibited an 81% higher risk of CLD (OR = 1.81, 95% CI = 1.32–2.46). Conversely, those with CLD had an 81% elevated risk of dyslipidemia (OR = 1.81, 95% CI = 1.33–2.46). Depressive symptoms mediated a statistically significant yet modest proportion of the bidirectional association (mediation proportions: 2.91% for the path from dyslipidemia to CLD; 2.54% for the path from CLD to dyslipidemia).

**Conclusion:**

A bidirectional relationship exists between dyslipidemia and CLD, partially mediated by depressive symptoms. While lipid regulation and CLD management are crucial, causal inferences are limited by the cross-sectional design. Future longitudinal or experimental studies are warranted to establish causality.

## 1 Introduction

With the rapid urbanization and changes in dietary patterns and lifestyles, the prevalence of dyslipidemia in China has significantly increased, making it a major risk factor for atherosclerotic cardiovascular diseases (1). Dyslipidemia is a general term encompassing all lipid metabolism disorders. Key lipid parameters frequently assessed in clinical practice include total cholesterol (TC), triglycerides (TG), low-density lipoprotein cholesterol (LDL-C), high-density lipoprotein cholesterol (HDL-C), and, more recently, lipoprotein(a) (2). According to the Global Burden of Disease Study 2019 (3), elevated LDL-C levels have risen from the 14th leading cause of death globally in 1990 to the 8th in 2019. LDL-C-related attributable risk is especially significant among older populations, ranking sixth in individuals aged 50–74 years, and fifth among those aged 75 years and older. With China rapidly transitioning into an aging society, this trend underscores an urgent need for enhanced prevention and management strategies for dyslipidemia and its associated diseases. A meta-analysis has reported that the overall prevalence of dyslipidemia among older adults in China is as high as 47.0% (4), providing critical evidence for the development of public health policies aimed at reducing disease burden.

Similarly, chronic liver diseases (CLDs), including viral hepatitis, alcoholic hepatitis, and metabolic dysfunction-associated steatohepatitis (MASH), represent leading causes of liver cirrhosis and hepatocellular carcinoma. These conditions are also major contributors to global morbidity and mortality (5). Epidemiological studies estimate that approximately 260 million individuals worldwide are living with chronic hepatitis B virus (HBV) infection (6), which has been recognized as a severe global public health challenge (7). Therefore, identifying risk factors and developing effective interventions for CLDs, particularly among middle-aged and older adult populations in China, is of paramount importance for mitigating disease burden and improving public health outcomes.

Dyslipidemia and its relationship with CLD have been a focus of considerable research. Evidence suggests that dyslipidemia is associated with an increased risk of liver disease events induced by hepatitis viruses (8). Conversely, several studies have shown that serum levels of TC and TG are reduced in patients with CLD (9, 10). CLD patients have higher HDL-C levels than healthy people (11). This reduction may be attributed to impaired hepatic cholesterol production due to liver dysfunction (12). However, the association between dyslipidemia and non-fatty liver CLD remains unclear, and its underlying mechanisms remain poorly understood. Depression, a common mental disorder among older adults, represents a significant public health challenge, further exacerbated by the COVID-19 pandemic. Globally, 7% of older adults are diagnosed with major depressive disorder. Depression in older adult populations is often complicated by comorbidities and physical illnesses, leading to increased disability, mortality, and healthcare burdens (13). Notably, depression is highly comorbid with dyslipidemia and CLD. For example, depressive symptoms frequently coexist with dyslipidemia (14). Dyslipidemia is often accompanied by abdominal obesity and chronic inflammation, which can influence neural and emotional regulation, thereby contributing to depressive disorders (15). Furthermore, adults with depression exhibit specific lipid profile changes, including decreased HDL-C levels and increased LDL-C and triglyceride levels (16, 17). Depression also independently increases the risk of hepatitis, with a hazard ratio of 1.17 in the general population (18). Among untreated CLD patients, 36.6 and 40% experience depression and anxiety, respectively (19). Based on this body of evidence, we hypothesize that depressive symptoms may serve as a potential mechanistic link between dyslipidemia and CLD. This study aims to: 1. Investigate the bidirectional relationship between dyslipidemia and CLD in China’s older adult populations. 2. Examine whether depressive symptoms mediate the relationship between dyslipidemia and CLD. Although previous longitudinal studies have confirmed the association between dyslipidemia and CLD, there is a lack of data elucidating the underlying mechanisms. Given the high prevalence of CLD and dyslipidemia in China, analyzing the mediating role of depressive symptoms in their interrelationship is crucial. This research holds the promise of providing essential insights for developing early prevention strategies.

## 2 Methods

### 2.1 Study sample

The China Health and Retirement Longitudinal Study (CHARLS) is a national, biennial longitudinal survey designed to advance multidisciplinary research on aging. Initiated between June 2011 and March 2012, the study employed a multistage, stratified probability sampling method with probability proportional to size. A total of 17,708 participants were recruited from 150 counties/districts and 450 villages across 28 provinces in China. The respondents have been followed up every 2 years, with five waves of comprehensive data collected so far (2011, 2013, 2015, 2018 and 2020). Standardized, face-to-face interviews were conducted to collect information on demographics, health status and functioning, socioeconomic characteristics, and retirement-related factors. The response rate for the first wave was 80.5% (20). This study utilized data from the 2015 wave of CHARLS. The baseline dataset included 21,095 participants, from which we excluded those with missing or abnormal data (*n* = 14,097), leaving 6,926 older adults eligible for analysis. Ethics approval for CHARLS was granted by the Institutional Review Board at Peking University (IRB00001052-11015). All participants provided written informed consent for both study participation and data sharing after being thoroughly informed of the study’s risks and benefits. Fasting venous blood samples were collected from each participant. Following centrifugation and processing, plasma aliquots were transported to KingMed Diagnostics Laboratory for testing, including high-sensitivity C-reactive protein (CRP), TG, and glucose levels. Additionally, body weight and height were measured using a standardized scale to 0.1 kg and 0.1 cm, respectively, with participants asked to remove shoes and heavy clothing. Detailed protocols for blood collection and biomarker assays have been described previously (21).

### 2.2 CLD

The diagnosis of CLD was based on participants’ self-reports of physician-diagnosed conditions during their medical visits. CLD included viral hepatitis, autoimmune hepatitis, primary biliary cholangitis, and primary sclerosing cholangitis, but excluded fatty liver disease, tumors, and cancer. To reduce potential recall bias, participants were additionally asked whether they were “currently receiving any treatment for chronic liver disease or its complications, such as traditional Chinese medicine, Western medicine, or other therapies.” The responses to these two questions were combined to classify CLD status as “Yes” or “No” (22).

### 2.3 Dyslipidemia

The classification of dyslipidemia was based on participants’ self-reported diagnosis by a physician or objective criteria: TC ≥ 240 mg/dL, TG ≥ 150 mg/dL, HDL-C < 40 mg/dL, or LDL-C ≥ 160 mg/dL. To minimize recall bias, participants were also asked whether they were “currently receiving any treatment for dyslipidemia or its complications, such as traditional Chinese medicine, Western medicine, or other therapies.” Combining self-reported answers and biochemistry data, participants’ dyslipidemia status was categorized as “Yes” or “No” (23).

### 2.4 Depressive symptoms

Depressive symptoms were assessed using the Center for Epidemiologic Studies Depression Scale (CES-D), a validated tool designed to evaluate the frequency of depressive symptoms experienced over the past week. Responses for each item were classified into four levels: “Rarely or none,” “Some or a little,” “Occasionally or moderate,” and “Most or all the time.” The CES-D includes 10 items: 1. being bothered by things that usually do not trouble you, 2. difficulty keeping your mind focused, 3. feeling depressed, 4. feeling everything you do is an effort, 5. feeling hopeful about the future, 6. feeling fearful, 7. restless sleep, 8. feeling happy, 9. feeling lonely, and 10. feeling like you cannot move forward. Before computing the total score, items 5 and 8 were reverse-coded. Each item was scored 0, 1, 2, or 3. A higher total CES-D score indicated greater severity of depressive symptoms. A CES-D score ≥ 10 was categorized as “depressive symptoms present,” indicating an increased risk of depression, while a score < 10 suggested the absence of significant depressive symptoms (24). Depressive symptoms were categorized as follows: none (0–9 points), mild (10–20 points), and moderate/severe (21–30 points) (25).

### 2.5 Covariates

The study included a range of sociodemographic and health-related covariates to provide a comprehensive analysis. These covariates were: age; sex/gender (female or male); educational attainment (categorized as illiterate, primary school, middle school, high school, or higher education); residence (urban or rural); marital status (married or single); body mass index (BMI); CRP levels; hypertension status, smoking status, drinking history, cancer, chronic lung disease; stroke, and diabetes (all coded as Yes or No). The triglyceride-glucose (TyG) index has been proposed as a robust marker of insulin resistance (IR). The TyG index is calculated as ln (fasting TG [mg/dL] × fasting blood glucose [mg/dL] / 2) (26). Life satisfaction was assessed through a single-item question: “How satisfied are you with your overall life satisfaction?” This item has been shown to correlate strongly with the validated 5-item Satisfaction with Life Scale and has been widely used in previous research. Response options ranged from “completely dissatisfied” to “completely satisfied” on a 5-point Likert scale, scored from 0 to 4, with higher scores indicating greater life satisfaction (27). Sleep duration was defined as the average number of hours participants reported falling asleep each night over the past month. Social activities were measured by asking participants: “Have you participated in the following social activities in the past month?” The activities included 11 categories: 1. interacting with friends, 2. playing games such as mahjong or chess or visiting community clubs, 3. assisting family, friends, or neighbors not living in the same household, 4. participating in sports, social, or club activities (e.g., dancing, fitness training, or qigong), 5. engaging in community-related organizations, 6. volunteer or charity work, 7. caregiving for patients or disabled adults not living with the participant, 8. attending education or training courses, 9. stock investment activities, 10. using the internet, and 11. other unspecified activities. Participants who engaged in any of these activities during the past month were coded as “Yes,” while those who did not participate in any were coded as “No.”

### 2.6 Statistical analysis

First, continuous variables were summarized as means standard deviations (SD), while categorical variables were reported as frequencies and percentages. Second, logistic regression analysis was performed to evaluate associations between primary variables. Following the mediation model proposed by Baron and Kenny (28), we tested whether depressive symptoms mediated the association between dyslipidemia and CLD: Logistic regression models were used to identify the bidirectional associations between dyslipidemia and CLD.

Depressive symptoms were then introduced to evaluate mediation effects. In model 1, the association between dyslipidemia and CLD mediated by depressive symptoms was adjusted for gender, age, marital status, educational attainment, and residence. In model 2, additional adjustments were made for diabetes, hypertension, BMI, cancer, chronic lung disease, stroke, smoking status, drinking history, and life satisfaction, TyG index, CRP, sleep duration, social activities. The fully adjusted model was then stratified by depressive symptom subgroups to explore the bidirectional relationship between dyslipidemia and CLD.

Subgroup analyses were conducted to compare the associations across different demographic groups (e.g., gender, age, education level, residence, and marital status, smoking status, and drinking history). To ensure robustness, we used a non-parametric bootstrap method with 1,000 resamples to estimate the total, indirect, and direct effects. For all logistic regression analyses, regression coefficients and *p* were reported. Mediation significance required *p* < 0.05.

To address the influence of missing data, we conducted sensitivity analyses by 1. excluding cases with extreme BMI or CRP values, 2. excluding participants with other chronic conditions (e.g., diabetes, hypertension, cancer, chronic lung disease, or stroke), 3. excluding participants with memory impairment (e.g., Alzheimer’s disease, brain atrophy, or Parkinson’s disease) to mitigate potential recall bias. All statistical analyses were performed using R software (version 4.2.2; R Foundation for Statistical Computing). *p* < 0.05 was considered statistically significant.

## 3 Results

### 3.1 Baseline characteristics of participants

The baseline characteristics are detailed in Table 1. A total of 6,926 individuals were included in the cohort, of whom 6,704 were categorized into the non-chronic liver disease (Non-CLD) group and 222 into the CLD group. The participants had a median age of 58.7 years (SD = 9.5), with 1,626 (23.5%) males and 5,300 (76.5%) females. Overall, 78.3% completed high school or higher education, 88.2% were married, and 81.4% resided in rural areas. The average BMI was 24.5 kg/m^2^ (SD = 4.0), and the mean TyG index was 8.8 (SD = 0.6). Significant differences were observed between the CLD and Non-CLD groups: CLD patients exhibited higher rates of dyslipidemia, smoking, chronic lung disease, and depressive symptoms, as well as lower levels of life satisfaction and shorter sleep duration, but higher participation in social activities (all *p* < 0.05). Additionally, 5,043 participants had no dyslipidemia versus 1,883 with dyslipidemia (Table 2). The dyslipidemia group displayed distinct characteristics: they were older, had a higher proportion of males, were more likely to have a high school education, and were more likely to reside in urban areas. They also exhibited higher smoking rates, elevated BMI, TyG index, and CRP levels. Furthermore, participants in the dyslipidemia group had increased prevalence of comorbidities, including CLD, hypertension, cancer, chronic lung disease, stroke, diabetes, and depressive symptoms, along with significantly greater engagement in social activities (all *p* < 0.05).

**Table 1:** Baseline characteristics of study population by CLD status.

Characteristics	Total (*n* = 6,926)	Non-CLD (*n* = 6,704)	CLD (*n* = 222)	*p*
Age, mean (SD)	58.7 (9.5)	58.7 (9.5)	58.1 (8.6)	0.391
Gender, *n* (%)				0.031
Female	5,300 (76.5)	5,144 (76.7)	156 (70.3)	
Male	1,626 (23.5)	1,560 (23.3)	66 (29.7)	
Education, *n* (%)				0.894
High school+	5,423 (78.3)	5,248 (78.3)	175 (78.8)	
Illiterate	513 (7.4)	498 (7.4)	15 (6.8)	
Middle school	287 (4.1)	276 (4.1)	11 (5.0)	
Primary	703 (10.2)	682 (10.2)	21 (9.5)	
Marital, *n* (%)				0.312
Married	6,106 (88.2)	5,905 (88.1)	201 (90.5)	
Single	820 (11.8)	799 (11.9)	21 (9.5)	
Residence, *n* (%)				1.000
Rural	5,641 (81.4)	5,460 (81.4)	181 (81.5)	
Urban	1,285 (18.6)	1,244 (18.6)	41 (18.5)	
BMI, mean (SD)	24.5 (4.0)	24.5 (4.0)	24.7 (3.8)	0.424
Dyslipidemia, *n* (%)				<0.001
No	5,043 (72.8)	4,908 (73.2)	135 (60.8)	
Yes	1883 (27.2)	1796 (26.8)	87 (39.2)	
Hypertension, *n* (%)				0.104
No	5,116 (73.9)	4,963 (74.0)	153 (68.9)	
Yes	1810 (26.1)	1741 (26.0)	69 (31.1)	
Cancer, *n* (%)				0.454
No	6,852 (98.9)	6,634 (99.0)	218 (98.2)	
Yes	74 (1.1)	70 (1.0)	4 (1.8)	
Chronic lung disease, *n* (%)				<0.001
No	6,442 (93.0)	6,255 (93.3)	187 (84.2)	
Yes	484 (7.0)	449 (6.7)	35 (15.8)	
Stroke, *n* (%)				0.727
No	6,835 (98.7)	6,617 (98.7)	218 (98.2)	
Yes	91 (1.3)	87 (1.3)	4 (1.8)	
Diabetes, *n* (%)				0.121
No	6,410 (92.5)	6,211 (92.6)	199 (89.6)	
Yes	516 (7.5)	493 (7.4)	23 (10.4)	
Depressive symptoms, *n* (%)				<0.001
No	4,493 (64.9)	4,380 (65.3)	113 (50.9)	
Yes	2,433 (35.1)	2,324 (34.7)	109 (49.1)	
Depressive symptoms subgroups, *n* (%)				<0.001
None	4,493 (64.9)	4,380 (65.3)	113 (50.9)	
Mild	1913 (27.6)	1829 (27.3)	84 (37.8)	
Moderate/Severe	520 (7.5)	495 (7.4)	25 (11.3)	
Smoking status, *n* (%)				0.038
No	6,176 (89.2)	5,988 (89.3)	188 (84.7)	
Yes	750 (10.8)	716 (10.7)	34 (15.3)	
Drinking history, *n* (%)				0.207
No	5,754 (83.1)	5,577 (83.2)	177 (79.7)	
Yes	1,172 (16.9)	1,127 (16.8)	45 (20.3)	
TyG index, mean (SD)	8.8 (0.6)	8.8 (0.6)	8.7 (0.6)	0.400
CRP, mean (SD)	2.5 (5.5)	2.5 (5.6)	2.3 (3.2)	0.603
Life satisfaction, mean (SD)	2.4 (0.8)	2.4 (0.8)	2.3 (0.9)	0.007
Sleep duration, mean (SD)	6.3 (1.9)	6.4 (1.9)	5.9 (1.9)	0.002
Social activities, *n* (%)				0.008
No	2,987 (43.1)	2,911 (43.4)	76 (34.2)	
Yes	3,939 (56.9)	3,793 (56.6)	146 (65.8)	

*p*-values were calculated using chi-square tests (for categorical variables), Wilcoxon rank-sum tests (for non-normally distributed continuous variables), and Student’s *t*-tests (for normally distributed continuous variables). CLD, chronic liver disease; TyG, triglyceride-glucose; CRP, c-reactive protein; SD, standard deviations; BMI, body mass index.

**Table 2:** Baseline characteristics of study population by dyslipidemia status.

Characteristics	Total (*n* = 6,926)	Non-dyslipidemia (*n* = 5,043)	Dyslipidemia (*n* = 1883)	*p*
Age, mean (SD)	58.7 (9.5)	58.5 (9.5)	59.2 (9.3)	0.004
Gender, *n* (%)				<0.001
Female	5,300 (76.5)	3,917 (77.7)	1,383 (73.4)	
Male	1,626 (23.5)	1,126 (22.3)	500 (26.6)	
Education, *n* (%)				<0.001
High school+	5,423 (78.3)	3,893 (77.2)	1,530 (81.3)	
Illiterate	513 (7.4)	407 (8.1)	106 (5.6)	
Middle school	287 (4.1)	204 (4.0)	83 (4.4)	
Primary	703 (10.2)	539 (10.7)	164 (8.7)	
Marital, *n* (%)				0.591
Married	6,106 (88.2)	4,439 (88.0)	1,667 (88.5)	
Single	820 (11.8)	604 (12.0)	216 (11.5)	
Residence, *n* (%)				<0.001
Rural	5,641 (81.4)	4,211 (83.5)	1,430 (75.9)	
Urban	1,285 (18.6)	832 (16.5)	453 (24.1)	
BMI, mean (SD)	24.5 (4.0)	24.1 (4.0)	25.7 (3.9)	<0.001
CLD, *n* (%)				<0.001
No	6,704 (96.8)	4,908 (97.3)	1796 (95.4)	
Yes	222 (3.2)	135 (2.7)	87 (4.6)	
Hypertension, *n* (%)				<0.001
No	5,116 (73.9)	3,979 (78.9)	1,137 (60.4)	
Yes	1810 (26.1)	1,064 (21.1)	746 (39.6)	
Cancer, *n* (%)				0.028
No	6,852 (98.9)	4,998 (99.1)	1854 (98.5)	
Yes	74 (1.1)	45 (0.9)	29 (1.5)	
Chronic lung disease, *n* (%)				0.006
No	6,442 (93.0)	4,717 (93.5)	1725 (91.6)	
Yes	484 (7.0)	326 (6.5)	158 (8.4)	
Stroke, *n* (%)				<0.001
No	6,835 (98.7)	4,999 (99.1)	1836 (97.5)	
Yes	91 (1.3)	44 (0.9)	47 (2.5)	
Diabetes, *n* (%)				<0.001
No	6,410 (92.5)	4,821 (95.6)	1,589 (84.4)	
Yes	516 (7.5)	222 (4.4)	294 (15.6)	
Depressive symptoms, *n* (%)				0.011
No	4,493 (64.9)	3,317 (65.8)	1,176 (62.5)	
Yes	2,433 (35.1)	1726 (34.2)	707 (37.5)	
Depressive symptoms subgroups, *n* (%)				0.036
None	4,493 (64.9)	3,317 (65.8)	1,176 (62.5)	
Mild	1913 (27.6)	1,357 (26.9)	556 (29.5)	
Moderate/Severe	520 (7.5)	369 (7.3)	151 (8.0)	
Smoking status, *n* (%)				0.016
No	6,176 (89.2)	4,525 (89.7)	1,651 (87.7)	
Yes	750 (10.8)	518 (10.3)	232 (12.3)	
Drinking history, *n* (%)				0.025
No	5,754 (83.1)	4,158 (82.5)	1,596 (84.8)	
Yes	1,172 (16.9)	885 (17.5)	287 (15.2)	
TyG index, mean (SD)	8.8 (0.6)	8.6 (0.6)	9.1 (0.7)	<0.001
CRP, mean (SD)	2.5 (5.5)	2.1 (4.3)	3.7 (7.7)	<0.001
Life satisfaction, mean (SD)	2.4 (0.8)	2.4 (0.8)	2.4 (0.8)	0.451
Sleep duration, mean (SD)	6.3 (1.9)	6.4 (1.9)	6.3 (1.9)	0.137
Social activities, *n* (%)				<0.001
No	2,987 (43.1)	2,252 (44.7)	735 (39.0)	
Yes	3,939 (56.9)	2,791 (55.3)	1,148 (61.0)	

*p*-values were calculated using chi-square tests (for categorical variables), Wilcoxon rank-sum tests (for non-normally distributed continuous variables), and Student’s *t*-tests (for normally distributed continuous variables). CLD, chronic liver disease; TyG, triglyceride-glucose; CRP, c-reactive protein; SD, standard deviations; BMI, body mass index.

### 3.2 Association between dyslipidemia and CLD in different models

Logistic regression analysis examined the associations between dyslipidemia, depressive symptoms, and CLD, with adjusted odds ratios (OR) and corresponding 95% confidence intervals (CI) calculated. As shown in Table 3, among middle-aged adults, dyslipidemia (OR = 1.81, 95% CI = 1.32–2.46, *p* < 0.001) and depressive symptoms (OR = 1.65, 95% CI = 1.22–2.22, *p* = 0.001) were significantly associated with increased CLD risk in fully adjusted models.

**Table 3:** Association between dyslipidemia and CLD in different models.

Variables	Model 1		Model 2	
	OR (95% CI)	*p*	OR (95% CI)	*p*
Dyslipidemia	1.69 (1.28, 2.23)	<0.001	1.81 (1.32, 2.46)	<0.001
Depressive symptoms	1.96 (1.48, 2.58)	<0.001	1.65 (1.22, 2.22)	0.001

Model 1 was adjusted for gender, age, marital status, education and residence. Model 2 was adjusted for gender, age, marital status, education, and residence, diabetes, hypertension, BMI, cancer, chronic lung disease, stroke, smoking status, drinking history, life satisfaction, TyG index, CRP, sleep duration and social activities. CI, confidence interval; OR, odds ratios; CLD, Chronic liver disease.

### 3.3 Association between CLD and dyslipidemia in different models

When CLD was treated as the independent variable (Table 4), it was significantly associated with dyslipidemia (OR = 1.81, 95% CI = 1.33–2.46, *p* < 0.001). Depressive symptoms also increased dyslipidemia risk (OR = 1.20, 95% CI = 1.05–1.37, *p* = 0.007) in fully adjusted models.

**Table 4:** Association between CLD and dyslipidemia in different models.

Variables	Model 1		Model 2	
	OR (95% CI)	*p*	OR (95% CI)	*p*
CLD	1.69 (1.28, 2.23)	<0.001	1.81 (1.33, 2.46)	<0.001
Depressive symptoms	1.26 (1.12, 1.41)	<0.001	1.20 (1.05, 1.37)	0.007

Model 1 was adjusted for gender, age, marital status, education and residence. Model 2 was adjusted for gender, age, marital status, education, and residence, diabetes, hypertension, BMI, cancer, chronic lung disease, stroke, smoking status, drinking history, life satisfaction, TyG index, CRP, sleep duration and social activities. CI, confidence interval; OR, odds ratios; CLD, Chronic liver disease.

### 3.4 Association between dyslipidemia and CLD stratified by depressive symptoms

As illustrated in Table 5, among participants without depressive symptoms, dyslipidemia was associated with a 72% higher CLD risk (OR = 1.72, 95% CI = 1.11–2.65, *p* = 0.015). In those with mild depressive symptoms, dyslipidemia was associated with a 74% higher risk increased CLD risk (OR = 1.74, 95% CI = 1.03–2.91, *p* = 0.036). The association was strongest in the moderate/severe depressive symptoms group (OR = 2.86, 95% CI = 1.06–7.70, *p* = 0.036). Conversely, when CLD was treated as the independent variable and dyslipidemia as the outcome, CLD significantly predicted dyslipidemia in participants without depressive symptoms (OR = 1.77, 95% CI = 1.14–2.71, *p* = 0.010) and with mild depressive symptoms (OR = 1.71, 95% CI = 1.02–2.82, *p* = 0.038). However, in the moderate/severe depressive symptoms group, CLD did not significantly increase dyslipidemia risk despite a trend toward association (OR = 2.45, 95% CI = 0.94–6.30, *p* = 0.063).

**Table 5:** Association between CLD and dyslipidemia stratified by depressive symptoms.

Depressive symptom Subgroups	Dyslipidemia-CLD		CLD-dyslipidemia	
	OR (95% CI)	*p*	OR (95% CI)	*p*
None	1.72 (1.11, 2.65)	0.015	1.77 (1.14, 2.71)	0.010
Mild	1.74 (1.03, 2.91)	0.036	1.71 (1.02, 2.82)	0.038
Moderate/Severe	2.86 (1.06, 7.70)	0.036	2.45 (0.94, 6.30)	0.063

Model was adjusted for gender, age, marital status, education, and residence, diabetes, hypertension, BMI, cancer, chronic lung disease, stroke, smoking status, drinking history, life satisfaction, TyG index, CRP, sleep duration and social activities. CI, confidence interval; OR, odds ratios; CLD, Chronic liver disease.

### 3.5 Mediating effect of depressive symptoms on the dyslipidemia and CLD

**Figure 1:**
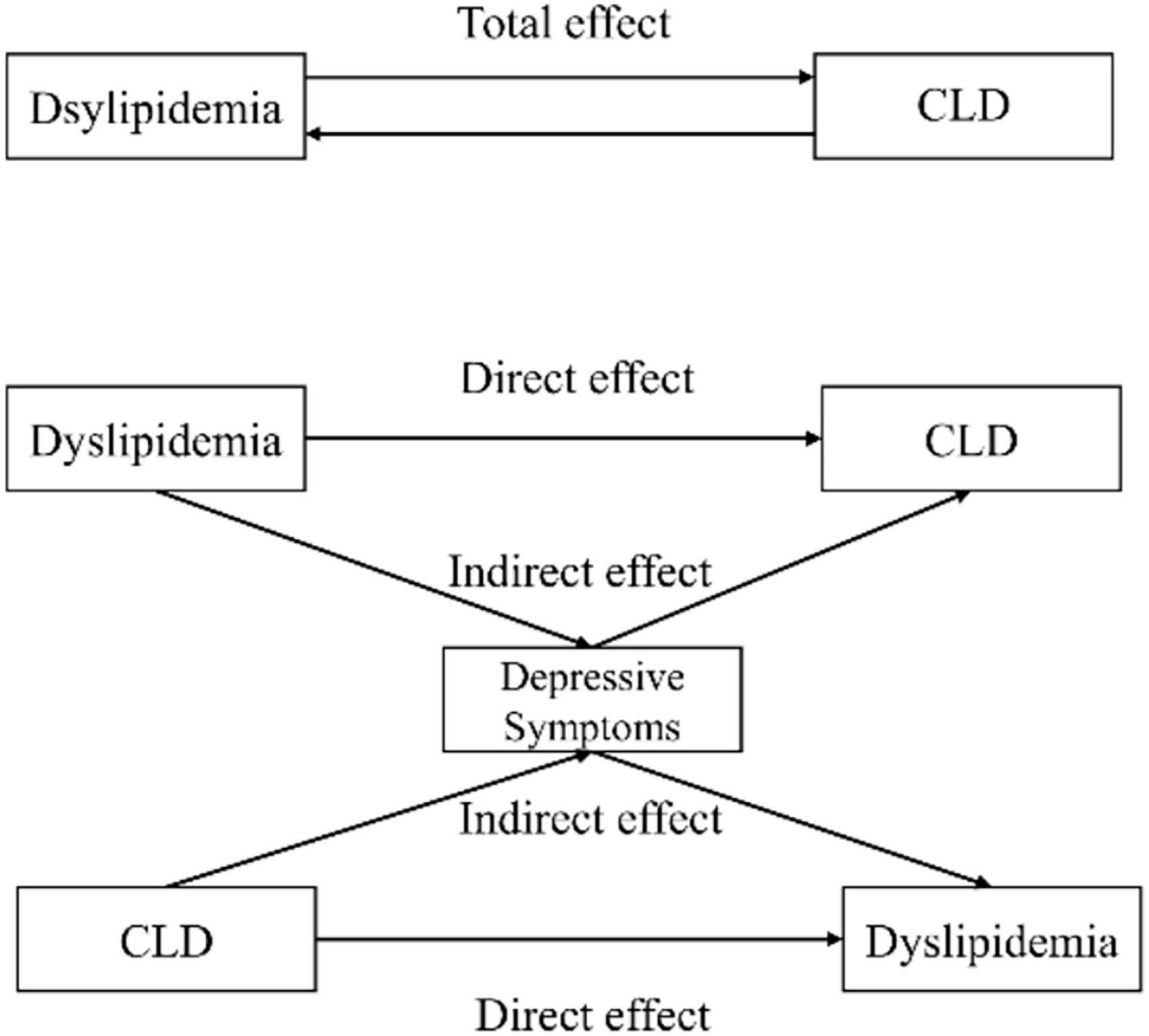
Path diagram of the median analysis models.

Mediation analysis (Table 6) revealed that depressive symptoms mediated 2.91% (*p* = 0.018) of the association between dyslipidemia and CLD. Conversely, when CLD was the independent variable and dyslipidemia the outcome, depressive symptoms explained 2.54% (*p* = 0.006) of the association. The conceptual mediation model is illustrated in Figure 1.

**Table 6:** The mediating effect of depressive symptoms on dyslipidemia and CLD.

Statistical Measure	Dyslipidemia-depressive symptoms-CLD		CLD-depressive symptoms-dyslipidemia	
	Estimate	*p*	Estimate	*p*
ACME (average)	0.00061	0.018	0.00272	0.006
ADE (average)	0.02023	<0.001	0.10456	<0.001
Prop. Mediated	0.02907	0.018	0.02535	0.006

CLD, Chronic liver disease; ADE, Average Direct Effect; ACME, Average Casual Mediation Effect. Multivariable-adjusted for were adjusted for gender, age, marital status, education, and residence, diabetes, hypertension, BMI, cancer, chronic lung disease, stroke, smoking status, drinking history, life satisfaction, TyG index, CRP, sleep duration, social activities.

### 3.6 Subgroups and sensitivity analyses

**Figure 2:**
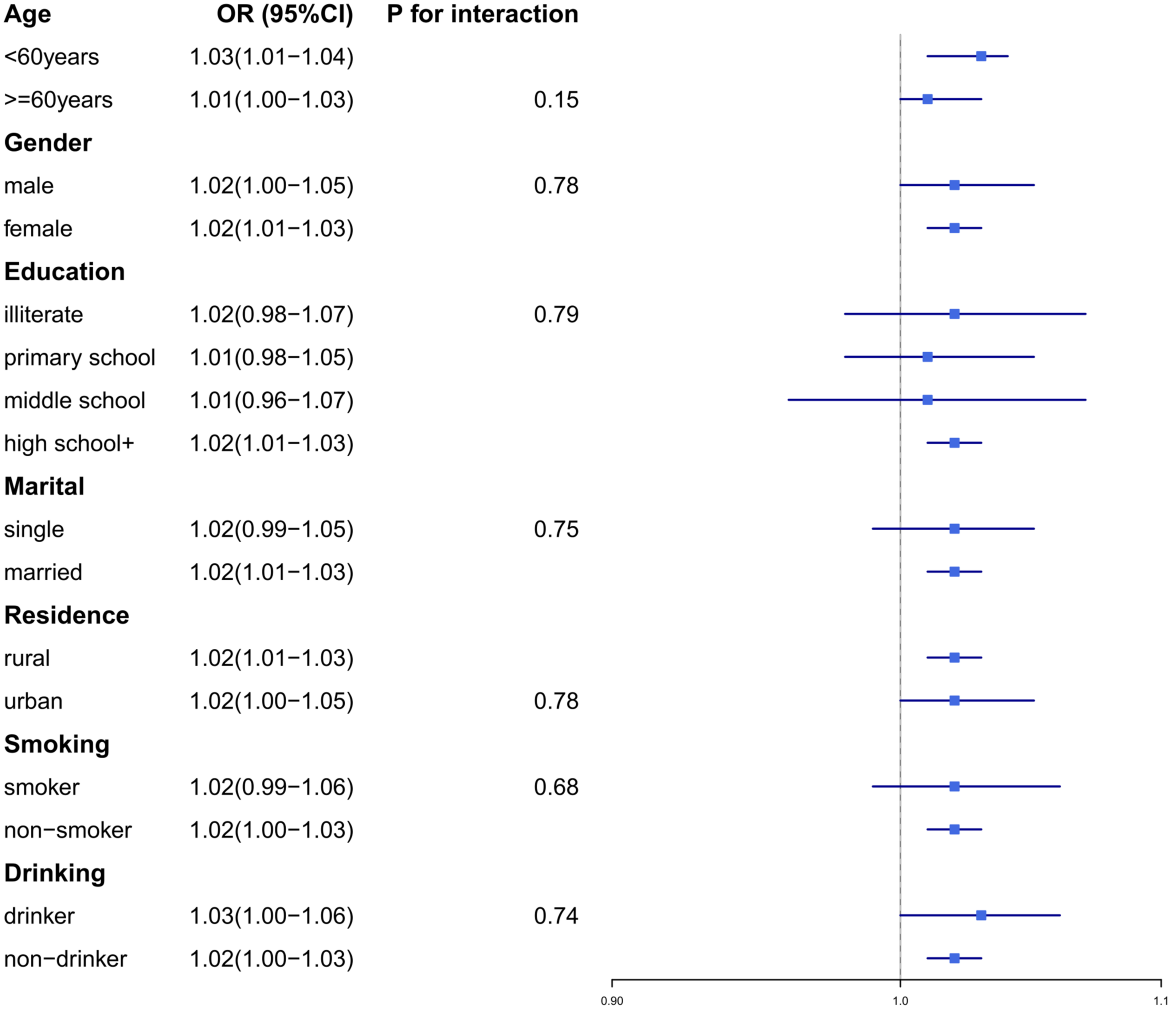
The associations between dyslipidemia and CLD differs among subgroups.

**Figure 3:**
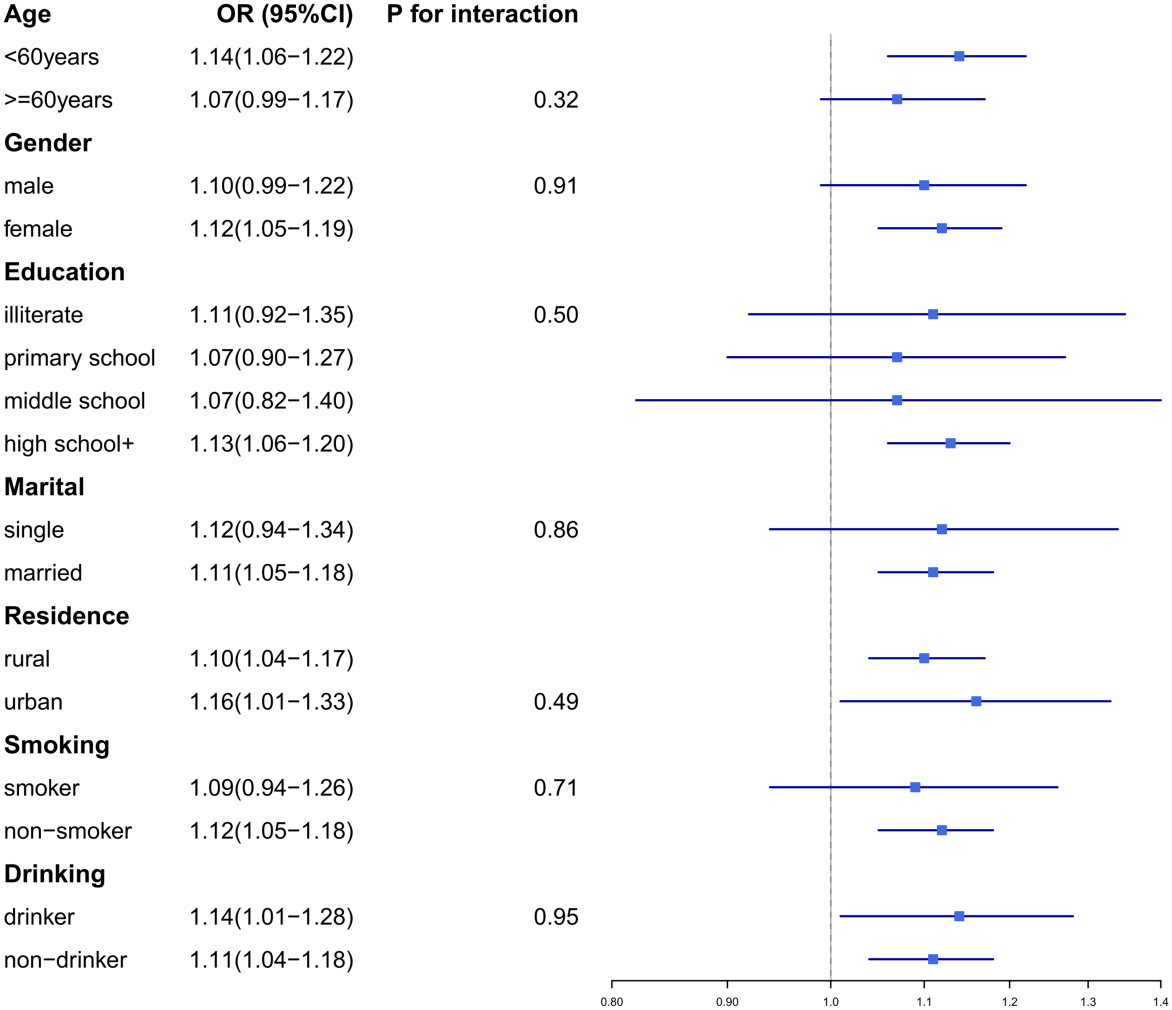
The associations between CLD and dyslipidemia differs among subgroups.

To validate the robustness of our findings, we conducted subgroup analyses and sensitivity analyses. Subgroup analyses were stratified by the following variables: age (≥60 years/<60 years), gender (male/female), education level (illiterate, primary school, middle school, or high school+), residence (urban/rural), marital status (married/single), smoking status (smoker/non-smoker), and drinking history (drinker/non-drinker) (Figures 2, 3). No significant interaction effects were observed across subgroups (*p* > 0.05). Additionally, the results remained consistent across multiple sensitivity analyses (Supplementary Tables 1–8).

## 4 Discussion

This study is the first to systematically investigate the potential bidirectional association between dyslipidemia and CLD in the Chinese older adult population. Our findings reveal a significant bidirectional positive correlation mediated by depressive symptoms. Globally, dyslipidemia and CLD represent substantial public health challenges, particularly in the Asia-Pacific region, which accounts for approximately half of CLD-related deaths, despite advancements in HBV vaccination and antiviral therapy, the COVID-19 pandemic has severely disrupted implementation efforts. As of 2022, the Asia-Pacific region accounts for approximately 1.6 million HBV-infected children under the age of five, representing 28.4% of the global burden in this demographic (29, 30). Vertical transmission and early-childhood infections remain key drivers of chronic HBV infections in this region. Therefore, exploring the mechanisms contributing to the increasing burden of CLD and dyslipidemia is urgently needed (31).

The interplay between dyslipidemia and CLD is not yet fully understood, though several studies suggest its participation in complex pathways, including IR, depression, and gut dysbiosis. Current evidence indicates that lipid deposition can significantly impair insulin sensitivity. Specifically, hepatic accumulation of diacylglycerol activates protein kinase C epsilon, which subsequently inhibits the tyrosine kinase activity of the insulin receptor, thereby worsening IR (32). This mechanism leads to impaired peripheral glucose uptake and may eventually progress to diabetes mellitus. IR concurrently intensifies hepatic lipid accumulation, further characterizing the progression of hepatitis (33). Hepatic steatosis can stimulate Kupffer cells to release inflammatory cytokines such as tumor necrosis factor-alpha (TNF-*α*) and interleukin-6 (IL-6), thus creating a vicious cycle that exacerbates both IR and dyslipidemia (34).

Our study demonstrates a significant independent bidirectional association between dyslipidemia and CLD, which persists after adjustment for demographic confounders (sex, age, marital status) and metabolic covariates including IR and CRP levels. Wang et al. identified HDL-C as an independent risk factor for HBV-related cirrhosis progression: patients with HDL-C levels <1.03 mmol/L had a 2.21-fold increased risk of cirrhosis (35). Mechanistically, hyperlipidemia induces a lipotoxic cascade by promoting intrahepatic deposition of circulating free fatty acids. This process primarily compromises mitochondrial function in hepatocytes and amplifies reactive oxygen species (ROS) generation, thereby inducing oxidative stress and endoplasmic reticulum stress, ultimately leading to hepatocyte inflammation and apoptosis (34). Conversely, CLD can also contribute to dyslipidemia. Research has demonstrated that HBV infection suppresses the secretion of hepatic very low-density lipoprotein (VLDL) via the HBx protein, leading to the accumulation of lipids within the liver (36).

Depression overlaps with dyslipidemia and CLD in pathophysiological mechanisms. Specifically, dyslipidemia and CLD exacerbate depressive symptoms through pathways involving the brain-gut-liver axis, chronic kidney disease-related mechanisms, and other factors (37, 38). Diets rich in saturated and monounsaturated fatty acids disrupt gut microbiota composition, promoting an overgrowth of lipopolysaccharide-producing bacteria and reducing short-chain fatty acid production, This consequently impairs vagal nerve modulation, while subsequent dysregulation of amino acid metabolism further inhibits serotonin (5-hydroxytryptamine, 5-HT) synthesis (39), while neurotransmitters such as acetylcholine, dopamine, and 5-HT directly regulate both depressive states and metabolic processes (40). A Taiwanese study revealed that the incidence of CLD in patients with bipolar disorder is 1.71 times higher than in the general population (41). Hepatic-derived pro-inflammatory factors penetrate the blood–brain barrier, directly inducing neuropathological changes (34). Patients with major depressive disorder exhibit significant abnormalities in bile acid metabolism, with a 2.27 times prevalence of CLD than non-depressed individuals (42). This may be attributed to bile acids acting on the central nervous system via direct or indirect pathway (43). Furthermore, depression synergizes with gut dysbiosis to hyperactivate the hypothalamic–pituitary–adrenal (HPA) axis, amplifying pro-inflammatory cytokine release and oxidative stress, which aggravates metabolic dysregulation (37). Depressive states also disrupt appetite and suppress intestinal motility, establishing a self-perpetuating vicious cycle.

Notably, when stratified by depression severity, our study revealed no significant association between dyslipidemia and CLD in the moderate/severe depression subgroup. This null finding may be attributable to the limited sample size within this subgroup. Furthermore, severe depressive symptoms likely exacerbate systemic inflammation and hormonal dysregulation (44), potentially driving the pathogenesis of acute vascular events (e.g., stroke or cardiovascular disease) rather than dyslipidemia. Consequently, clinical attention and diagnostic resources in this population are often prioritized toward managing these higher-acuity conditions, potentially obscuring associations with dyslipidemia.

Dyslipidemia and CLD form a bidirectional reinforcing network through multifaceted pathways, including metabolic disorders, viral interference, neuroendocrine dysregulation, and the microbiota-gut-brain axis. Depression manifests as both a consequence of this network and a driver of its progression. In our study, depressive symptoms played a statistically significant yet relatively minor mediating role in the bidirectional association between dyslipidemia and CLD (mediation proportions of 2.91 and 2.54%, respectively). Based on the aforementioned mechanisms and findings, we underscore the necessity of implementing comprehensive interventions to alleviate the disease burden of both conditions. Healthcare institutions should routinely monitor lipid profiles in older adults, particularly in high-risk populations, while prioritizing the interruption of viral hepatitis transmission and regularly following up with CLD patients. Psychological health management should be integrated into prevention strategies, with increased awareness of the influence of depression. Local governments and communities should promote anti-inflammatory diets (e.g., reduced red meat consumption) and physical activity to improve lipid metabolism, thereby reducing the risks of CLD and depression (45). Future research should consider additional factors, such as dietary habits or gut microbiota, and investigate their potential impact on the relationship between dyslipidemia and CLD. This exploration may reveal novel opportunities for intervention and prevention strategies. Our findings underscore the complexity of the dyslipidemia-CLD relationship and emphasize the critical need for ongoing research in this field.

Several limitations should be acknowledged in this study. First, the cross-sectional design may be insufficient to elucidate complex causal relationships between variables; prospective cohort studies are recommended to validate the longitudinal effects of depressive symptoms and dyslipidemia on CLD in middle-aged and older adult population. Second, although multidimensional questionnaires were used to comprehensively assess CLD, recall bias may persist; self-reported variables could lead to misclassification, necessitating integration of objective health monitoring data to improve accuracy in future research. Third, as findings are derived from a Chinese cohort aged ≥45 years, their generalizability to younger populations or other geographic regions may be limited; multicenter prospective studies are needed for broader validation. Fourth, dyslipidemia was assessed as a binary variable (based on integrated evaluation of blood tests, physician diagnosis, and lipid-lowering medication history); future studies should investigate its severity and subtypes in relation to CLD. Finally, specific associations between dyslipidemia and CLD of different etiologies (e.g., viral hepatitis, fatty liver disease) warrant focused attention.

## 5 Conclusion

Utilizing nationally representative data from the 2015 CHARLS, this study confirms a bidirectional positive association between dyslipidemia and CLD among older Chinese adults. Notably, depressive symptoms exhibit a partial mediating role in this relationship. Implementing integrated interventions—such as regular physical activity, reduced intake of high-fat diets; and optimized management of depressive symptoms—may mitigate disease burden and improve health outcomes in this population.

## Data Availability

The original contributions presented in the study are included in the article/Supplementary material, further inquiries can be directed to the corresponding author.
