# Supplementary material for: The relationship between dyslipidemia and chronic liver disease, with the mediating role of depressive symptoms
Source: Front Public Health. 2025 Aug 25;13:1581622. doi: 10.3389/fpubh.2025.1581622 (PMC12414773; doi:10.3389/fpubh.2025.1581622)
Supplement: Supplementary file 1 [file Data_Sheet_1.docx]

| Variables | Sensitivity analyses 1 | |  | Sensitivity analyses 2 | |
| --- | --- | --- | --- | --- | --- |
|  | *OR* (95% *CI*) | *p* |  | *OR* (95% *CI*) | *p* |
| Dyslipidemia | 1.82 (1.33, 2.48) | <0.001 |  | 1.80 (1.30, 2.48) | <0.001 |
| Depressive symptoms | 1.66 (1.23, 2.25) | <0.001 |  | 1.60 (1.17, 2.18) | 0.003 |

Table 1 Sensitivity analyses for the combined association between dyslipidemia and CLD.

Sensitivity analyses 1: Exclude cases with extreme values of BMI. Sensitivity analyses 2: Exclude cases with extreme values of CRP. Sensitivity analyses 1 and sensitivity analyses 2 were adjusted for gender, age, marital status, education, and residence, diabetes, hypertension, BMI, cancer, chronic lung disease, stroke, smoking stuatus, drinking history, life satisfaction, TyG index, CRP, sleep duration and social activities. *CI*, confidence interval; *OR*, odds ratios; CLD, Chronic liver disease.

| Variables | Sensitivity analyses 1 | |  | Sensitivity analyses 2 | |
| --- | --- | --- | --- | --- | --- |
|  | *OR* (95% *CI*) | *p* |  | *OR* (95% *CI*) | *p* |
| CLD | 1.82 (1.33, 2.48) | <0.001 |  | 1.82 (1.31, 2.49) | <0.001 |
| Depressive symptoms | 1.21 (1.06, 1.38) | 0.006 |  | 1.18 (1.03, 1.36) | 0.019 |

Table 2 Sensitivity analyses for the combined association between CLD and dyslipidemia.

Sensitivity analyses 1: Exclude cases with extreme values of BMI. Sensitivity analyses 2: Exclude cases with extreme values of CRP. Sensitivity analyses 1 and sensitivity analyses 2 were adjusted for gender, age, marital status, education, and residence, diabetes, hypertension, BMI, cancer, chronic lung disease, stroke, smoking status, drinking history, life satisfaction, TyG index, CRP, sleep duration and social activities. *CI*, confidence interval; *OR*, odds ratios; CLD, Chronic liver disease.

| Variables | Sensitivity analyses 3 | |  | Sensitivity analyses 4 | |
| --- | --- | --- | --- | --- | --- |
|  | *OR* (95% *CI*) | *p* |  | *OR* (95% *CI*) | *p* |
| Dyslipidemia | 2.03 (1.30, 3.13) | 0.002 |  | 1.80 (1.31, 2.46) | <0.001 |
| Depressive symptoms | 1.70 (1.12, 2.58) | 0.012 |  | 1.63 (1.20, 2.21) | 0.002 |

Table 3 Sensitivity analyses for the combined association between dyslipidemia and CLD.

Sensitivity analyses 3: Exclude cases with other chronic diseases. Sensitivity analyses 4: Exclude cases with memory disorders. Sensitivity analyses 3 were adjusted for age, gender, education, residence, marital, BMI, smoking, drinking, life satisfaction, TyG index, CRP, sleep duration and social activities. Sensitivity analyses 4 were adjusted for gender, age, marital status, education, and residence, diabetes, hypertension, BMI, cancer, chronic lung disease, stroke, smoking status, drinking history, life satisfaction, TyG index, CRP, sleep duration and social activities. *CI*, confidence interval; *OR*, odds ratios; CLD, Chronic liver disease.

| Variables | Sensitivity analyses 3 | |  | Sensitivity analyses 4 | |
| --- | --- | --- | --- | --- | --- |
|  | *OR* (95% *CI*) | *p* |  | *OR* (95% *CI*) | *p* |
| CLD | 2.04 (1.31, 3.14) | 0.001 |  | 1.80 (1.31, 2.46) | <0.001 |
| Depressive symptoms | 1.24 (1.03, 1.48) | 0.020 |  | 1.20 (1.05, 1.38) | 0.007 |

Table 4 Sensitivity analyses for the combined association between CLD and dyslipidemia.

Sensitivity analyses 3: Exclude cases with other chronic diseases. Sensitivity analyses 4: Exclude cases with memory disorders. Sensitivity analyses 3 were adjusted for age, gender, education, residence, marital, BMI, smoking, drinking, life satisfaction, TyG index, CRP, sleep duration and social activities. Sensitivity analyses 4 were adjusted for gender, age, marital status, education, and residence, diabetes, hypertension, BMI, cancer, chronic lung disease, stroke, smoking status, drinking history, life satisfaction, TyG index, CRP, sleep duration and social activities. *CI*, confidence interval; *OR*, odds ratios; CLD, Chronic liver disease.

Table 5 Sensitivity Analysis 1: Mediating Effect of Depressive symptoms in the Association Between Dyslipidemia and CLD.

| Statistical Measure | Dyslipidemia-Depressive symptoms-CLD | |  | CLD-Depressive symptoms-Dyslipidemia | |
| --- | --- | --- | --- | --- | --- |
|  | Estimate | *p* |  | Estimate | *p* |
| ACME (average) | 0.00087 | 0.012 |  | 0.00359 | 0.016 |
| ADE (average) | 0.02040 | <0.001 |  | 0.10523 | <0.001 |
| Prop. Mediated | 0.04080 | 0.012 |  | 0.03302 | 0.016 |

Multivariable-adjusted for were adjusted for gender, age, marital status, education, and residence, diabetes, hypertension, BMI, cancer, chronic lung disease, stroke, smoking status, drinking history, life satisfaction, TyG index, CRP, sleep duration and social activities. CLD, Chronic liver disease; ADE, Average Direct Effect; ACME, Average Casual Mediation Effect.

Table 6 Sensitivity Analysis 2: Mediating Effect of Depressive symptoms in the Association Between Dyslipidemia and CLD.

| Statistical Measure | Dyslipidemia-Depressive symptoms-CLD | |  | CLD-Depressive symptoms-Dyslipidemia | |
| --- | --- | --- | --- | --- | --- |
|  | Estimate | *p* |  | Estimate | *p* |
| ACME (average) | 0.00095 | 0.032 |  | 0.00322 | 0.012 |
| ADE (average) | 0.02030 | <0.001 |  | 0.10397 | <0.001 |
| Prop. Mediated | 0.04470 | 0.032 |  | 0.03000 | 0.012 |

Multivariable-adjusted for were adjusted for gender, age, marital status, education, and residence, diabetes, hypertension, BMI, cancer, chronic lung disease, stroke, smoking status, drinking history, life satisfaction, TyG index, CRP, sleep duration and social activities. CLD, Chronic liver disease; ADE, Average Direct Effect; ACME, Average Casual Mediation Effect.

Table 7 Sensitivity Analysis 3: Mediating Effect of Depressive symptoms in the Association Between Dyslipidemia and CLD.

| Statistical Measure | Dyslipidemia-Depressive symptoms-CLD | |  | CLD-Depressive symptoms-Dyslipidemia | |
| --- | --- | --- | --- | --- | --- |
|  | Estimate | *p* |  | Estimate | *p* |
| ACME (average) | 0.00080 | 0.038 |  | 0.00455 | 0.040 |
| ADE (average) | 0.02050 | <0.001 |  | 0.11600 | 0.002 |
| Prop. Mediated | 0.03740 | 0.038 |  | 0.03770 | 0.042 |

Multivariable-adjusted for were adjusted for gender, age, marital status, education, and residence, BMI, smoking status, drinking history, life satisfaction, TyG index, CRP, sleep duration and social activities. CLD, Chronic liver disease; ADE, Average Direct Effect; ACME, Average Casual Mediation Effect.

Table 8 Sensitivity Analysis 4: Mediating Effect of Depressive symptoms in the Association Between Dyslipidemia and CLD.

| Statistical Measure | Dyslipidemia-Depressive symptoms-CLD | |  | CLD-Depressive symptoms-Dyslipidemia | |
| --- | --- | --- | --- | --- | --- |
|  | Estimate | *p* |  | Estimate | *p* |
| ACME (average) | 0.00071 | 0.012 |  | 0.00270 | 0.002 |
| ADE (average) | 0.01963 | <0.001 |  | 0.10317 | <0.001 |
| Prop. Mediated | 0.03476 | 0.012 |  | 0.02551 | 0.002 |

Multivariable-adjusted for were adjusted for gender, age, marital status, education, and residence, diabetes, hypertension, BMI, cancer, chronic lung disease, stroke, smoking status, drinking history, life satisfaction, TyG index, CRP, sleep duration and social activities. CLD, Chronic liver disease; ADE, Average Direct Effect; ACME, Average Casual Mediation Effect.
